# A Treatise on the Practice of Surgery

**Published:** 1857-01

**Authors:** 


ART. IX.—*A Treatise on the Practice of Surgery.* By HENRY H. SMITH, M. D., Professor of the Principles and Practice of Surgery in the University of Pennsylvania; Consulting Surgeon to the St. Joseph's Hospital, Philadelphia; author of a Treatise on Operative Surgery, etc., etc. Illustrated by 274 engravings on wood. Philadelphia: J. B. LIPPINCOTT & Co. 1856.

Most new incumbents of chairs in the Philadelphia schools, take to authorship as a part of their functions as teachers. Prof. Smith is the successor of Gibson, as the surgeon of the University of Pennsylvania. There was no immediate necessity for a new text-book on Surgery, but there was an immediate necessity that Prof. Smith should write a book. He has done so, and the result is before us.

Our New York teachers might profit pecuniarily by doing something of the same kind. Mott is hardly known as an author, his fame lies in what he has done, not in what he has said. Parker has a vicarious authorship as —a long time ago—the editor of Cooper's First Series. Bedford has written somewhat and declaimed more. Gilman is yet to be an author, though we learn that he is standing god-father to a new edition of Beck's Materia Medica. Clark, than whom no man is more competent to write a book which would be a positive addition to science, contents himself with an evening at the Pathological Society; in fact only rare sporadic cases of authorship, and those usually harmless, are known in New York, while in Philadelphia the disease is endemic, with a regular autumnal epidemic coming on at the subsidence of the annual yellow fever discussion, (is it contagious?) and just before the opening of lecture terms.

Necessarily a new book on surgery, if written by an intelligent and competent man, must contain some new facts or new interpretations of old ones; must represent the latest advance in pathological knowledge or the latest appliance of surgical skill. Very many of our surgical works still in the market and still relied upon as books of reference by the practitioner, were written before the advent of chloroform; before the microscope had become an adjuvant in diagnosis, or before those recent additions to the pathology of surgery, which are so important and should be made familiar to the practitioner. Even the most standard works on surgery have lamentable deficiencies. Chelius, the largest of them, is a triple wilderness of wisdom where facts of the highest value are thrown in, helter-skelter, in such a confusion of arrangement as defies the help of an index.

From these negative objections, Smith's Operative Surgery is free. Written recently, it is a book for the present time. It contains all those minor matters of improvement, those little aids which are in the aggregate so valuable, and, moreover, in the department of scientific research, though it has little claim to originality, it represents the progressive scholars. Paget's valuable additions to surgical pathology are given in a condensed and practical shape, and from the glance which we have been able to give to the work, we should consider it a fair representation of the present condition of surgical science. The wood cuts are very neat and descriptive, and the typography excellent.

For sale by PHINNEY & Co.

